# Supplementary material for: Farmers’ perception of the ecosystem services provided by diurnal raptors in arid Rajasthan
Source: PeerJ. 2023 Aug 23;11:e15996. doi: 10.7717/peerj.15996 (PMC10460152; doi:10.7717/peerj.15996)
Supplement: Supplemental Information 3 [file peerj-11-15996-s003.docx]

Farmers perception Questionnaire (To be filled by the interviewer)

1. Name of the respondent-

2. Place of Residence: a) District…………. b) Tehsil/Block…………. c) Village…………… d) GPS Coordinates :………….Latitude ………Longitude

3. List the crops you grow:

| Vegetable Crops | Forage/Fodder Crops | Fruit Crops | Seed Crops |
| --- | --- | --- | --- |
|  |  |  |  |

4. Tick the types of livestock you raise:

| Buffalo | Cow | Goat/Sheep | Camel |
| --- | --- | --- | --- |
|  |  |  |  |

5. Age: ……………

6.Gender:………….

7.Level of Education:

| Primary School | Senior Secondary | Higher Secondary | College | None |
| --- | --- | --- | --- | --- |
|  |  |  |  |  |

8. Do you use organic or conventional farming techniques? £ Organic Conventional Both

9. Do you practice poultry management? ….Yes. ….No

10. If yes, have you faced chicken predation by raptors? …..Yes. ….No

11.Do you think Raptors are beneficial to you on the basis of following criteria:

| Criteria | Strongly Disagree  (1) | Disagree  (2) | Neither beneficial nor Harmful  (3) | Agree  (4) | Strongly Agree  (5) |
| --- | --- | --- | --- | --- | --- |
| Increases crop quality |  |  |  |  |  |
| Increases crop yield |  |  |  |  |  |
| Essential for Crop Production |  |  |  |  |  |
| Causes damage to pollinators |  |  |  |  |  |
| Causes damage to poultry |  |  |  |  |  |
| Causes damage to livestock |  |  |  |  |  |
| Controls Insects |  |  |  |  |  |
| Controls Rodents |  |  |  |  |  |
| Alternative to Pesticides |  |  |  |  |  |

12. Of the Raptor species that you see in your area, did you see them more, less or equal as compared to past years: a). 1 (decreasing) b) 0 ( no change) c) 1( increasing)

13. What is your overall perception of Raptors: ……Harmful. ……Beneficial

14. Are you willing to spend for conservation of raptors? ….Yes. …..No

15. Have you applied any external measure to provide habitat for raptors in your farm field? …..Yes …….No

16. If yes, then explain what measures have you taken?

……..Plant habitat ………Provide food ………Provide water (i.e. flood fields) ……….. Erect Perches ……….Erect nest boxes ………………. Other (please specify):

17. What is your perception of bats on the following criteria?

| Criteria | Strongly Disagree  (1) | Disagree  (2) | Neither beneficial nor Harmful  (3) | Agree  (4) | Strongly Agree  (5) |
| --- | --- | --- | --- | --- | --- |
| Bats are helpful in Insect Control |  |  |  |  |  |
| Bats cause damage/Disease to Livestock/Animals |  |  |  |  |  |
| Bats cause damage/Disease to Humans |  |  |  |  |  |
| Bats are beneficial for Crop Yield |  |  |  |  |  |

18. What is your perception of perching birds on the following criteria?

| Criteria | Strongly Disagree  (1) | Disagree  (2) | Neither beneficial nor Harmful  (3) | Agree  (4) | Strongly Agree  (5) |
| --- | --- | --- | --- | --- | --- |
| Perching Birds are beneficial in Insect Control |  |  |  |  |  |
| Perching Birds cause damage/Disease to Livestock/Animals |  |  |  |  |  |
| Perching Birds are beneficial for tourism |  |  |  |  |  |
| Perching Birds are beneficial for Crop Yield |  |  |  |  |  |
| Perching Birds are beneficial as pollinators |  |  |  |  |  |
