# Supplementary material for: Farmers’ perception of the ecosystem services provided by diurnal raptors in arid Rajasthan
Source: PeerJ. 2023 Aug 23;11:e15996. doi: 10.7717/peerj.15996 (PMC10460152; doi:10.7717/peerj.15996)
Supplement: Supplemental Information 4 [file peerj-11-15996-s004.docx]

Supporting information S1: Details of Socio-Demographic data collected from respondents

| Gender | Age | Education | Farming Method | Choice of Crop | Chicken Ownership |
| --- | --- | --- | --- | --- | --- |
| Male(n=249) | 20-35 (n=89) | Primary (n=98) | Conventional (n=289) | Forage(n=151) | Yes(n=104) |
| Female(n=124) | 36-50(n=162) | Senior Secondary(n=85) | Organic (n=74) | Fruit(n=89) | No (n=269) |
|  | 50-65(n=93) | Higher Secondary(n=83) | Both (n=10) | Seed(n=224) |  |
|  | 65-80(n=29) | College(n=2) |  | Vegetables(n=239) |  |
|  |  | None(n=105) |  |  |  |
|  |  |  |  |  |  |

Supporting information S2: Details of Regression analysis to understand the relationship between perception and the trend of population

Residuals:

Min 1Q Median 3Q Max

-1.41377 -0.75168 0.01918 0.59890 1.46480

| Coefficients: | Estimate | Std. Error | t value | Pr(>\|t\|) |
| --- | --- | --- | --- | --- |
| (Intercept) | 0.63025 | 0.07612 | 8.280 | 5.73e-16 *** |
| Perception | -0.21648 | 0.02278 | -9.503 | < 2e-16 *** |
| SpecieVultures | -0.01267 | 0.05972 | -0.212 | 0.832 |
| Residual standard error: 0.794 on 743 degrees of freedom | | | | |
| Multiple R-squared: 0.1147, Adjusted R-squared: 0.1123 | | | | |
| F-statistic: 48.14 on 2 and 743 DF, p-value: < 2.2e-16 | | | | |

Signif. codes:

0 ‘***’ 0.001 ‘**’ 0.01 ‘*’ 0.05 ‘.’ 0.1 ‘ ’ 1

Supporting information S3: Scree plot (Male respondents) to estimate number of factors for broad categoarization of ecosystem categories


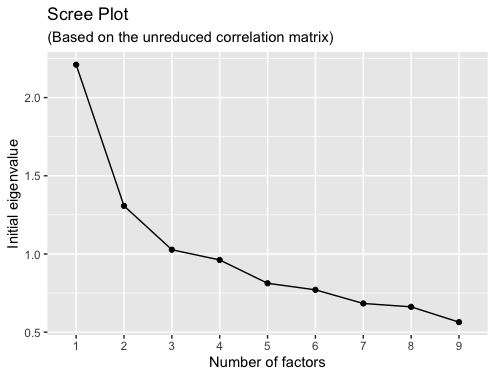


Supporting information S4: Scree plot (Male respondents) to estimate number of factors for broad categoarization of ecosystem categories


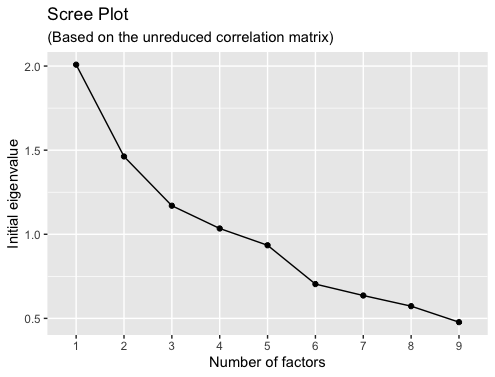


Supporting information S5: Perception of Male and Female Respondents on various factors towards Raptors : Mean (S.D.)

| Gender | Insect Control | Rodent Control | Crop Yield | Pollinators | Animals/Livestock | Tourism |
| --- | --- | --- | --- | --- | --- | --- |
| Male | 3.48(1.09) | 4.03(0.70) | 3.44 (1.23) | 3.13(1.42) | 2.59(1.28) | 3.56(1.34) |
| Female | 3.79(0.85) | 3.91(0.73) | 3.26(1.40) | 2.91(1.45) | 3.24(1.39) | 3.17(1.39) |

Supporting information S6: Perception of Male and Female Respondents on various factors towards Bats : Mean (S.D.)

| Gender | Insect Control | Rodent Control | Crop Yield | Pollinators | Animals/Livestock | Disease in Humans |
| --- | --- | --- | --- | --- | --- | --- |
| Male | 3.63(1.10) | NA | 3.00(1.35) | NA | 2.40(1.25) | 2.29(1.12) |
| Female | 3.79(1.12) | NA | 2.92(1.41) | NA | 3.24(2.40) | 2(0.89) |

Supporting information S7: Perception of Male and Female Respondents on various factors towards Perching Birds: Mean (S.D.)

| Gender | Insect Control | Rodent Control | Crop Yield | Pollinators | Animals/Livestock | Tourism |
| --- | --- | --- | --- | --- | --- | --- |
| Male | 3.12(1.27) | NA | 2.86(1.40) | 3.12(1.41) | 2.99(1.39) | 3.56(1.30) |
| Female | 3.27(1.04) | NA | 2.89(1.35) | 2.83(1.44) | 3.41(1.45) | 4.09 (1.10) |

Supporting information S8. Overall Perception of Bats,Perching Birds and Raptors among Respondents

| Species | Insect Control | Rodent Control | Crop Yield | Pollinators | Animals/Livestock | Disease in Humans | Tourism |
| --- | --- | --- | --- | --- | --- | --- | --- |
| Raptor | 3.58 (1.02) | 3.99(0.71) | 3.38 (1.29) | 3.06 (1.43) | 2.81(1.35) | NA | 3.43 (1.37) |
| Bat | 3.70 (1.11) | NA | 2.98 (1.37) | NA | 2.57(1.31) | 2.19 (1.06) | NA |
| Perching Birds | 3.17 (1.20) | NA | 2.87 (1.38) | 3.03 (1.43) | 3.13 (1.42) | NA | 3.74 (1.26) |

Supporting information S9. Perception of Male and Female respondents towards Bats.


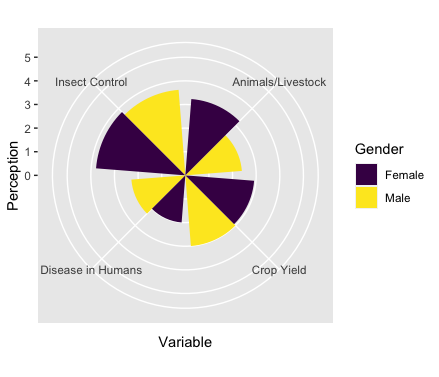


Supporting information S10. Perception of Male and Female respondents towards Perching Birds.


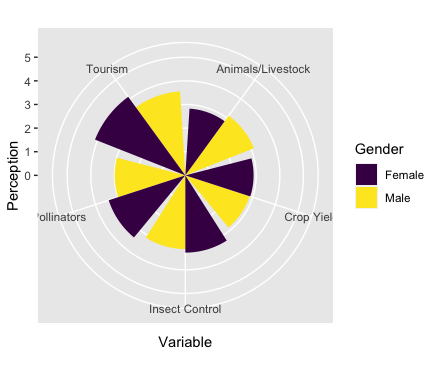


Supporting information S11: ANOVA statistics between perception of male and female respondents on Ecosystem Services: Mean (SD)

| Factor | Male Respondents Mean and SD | F- Ratio and p Value | Female Respondents Mean and SD |
| --- | --- | --- | --- |
| A1 | 3.82 (0.80) | 24.86 (p=.0007) | 4.11(1.16) |
| A2 | 3.67(0.77) | 0.03 (p<.852) | 3.83(1.09) |
| A3 | 3.74(0.67) | 24.17(p<.0001) | 4.15(0.87) |
| B1 | 1.74(0.63) | 2.95(p=.086) | 1.62(0.73) |
| B2 | 1.92(0.74) | .006(p=.804) | 1.94(1.04) |
| B3 | 1.84(0.74) | 0.0013(p=0.971) | 1.83(1.01) |
| C1 | 3.38(0.86) | 5.29(p=.021) | 3.60(0.93) |
| C2 | 3.45(0.85) | 6.09(p=.013) | 3.20(0.98) |
| C3 | 3.06(0.75) | 9.15(p=.002) | 3.35(1.06) |
